# Strengthening Primary Health Care for Improved Population Health and Health Equity in Somalia: A Narrative Review

**DOI:** 10.1002/puh2.70299

**Published:** 2026-06-28

**Authors:** Yusuf Abdullahi Hubow, Ilyas Abdullahi Khalif, Sharmake Gaiye Bashir, Ahmed Mohamed Omar, Narura Omar Mohamed, Ayan Abdullahi Mohammed, Hibo hassan Mohamed, Nour Ahmed Dahir, Aniso Mohamed Abdi, Abas Nor Abdi, Ahmed Abdinasir Abdulle

**Affiliations:** ^1^ Faculty of Health Sciences and Tropical Medicine Somali National University Mogadishu Somalia; ^2^ Center for Health Research and Innovation Somali National University Mogadishu Somalia; ^3^ Faculty of Health Science Salaam University Mogadishu Somalia

**Keywords:** health equity, population health, primary health care, Somalia, universal health coverage

## Abstract

Primary health care (PHC) is widely recognized as the foundation of equitable health systems and is a critical pathway towards universal health coverage and improved population health. In fragile and conflict‐affected settings, such as Somalia, weak PHC systems have contributed to persistently high maternal and child mortality, preventable infectious diseases, rising noncommunicable disease burdens, and profound health inequities affecting rural communities, internally displaced persons, women, and children. This narrative review examines how strengthening PHC can improve population health outcomes and advance health equity in Somalia. Drawing on peer‐reviewed literature, global PHC frameworks, and Somalia‐specific policy and health system evidence, this review synthesizes key challenges and opportunities across core PHC domains, including governance, financing, workforce development, service delivery, quality improvement, resilience, and community engagement. The findings highlight that fragmented governance, high out‐of‐pocket spending, workforce maldistribution, and limited rural and displacement‐sensitive service delivery constrain PHC performance and reinforce inequality. Simultaneously, recent policy reforms, community health worker programs, and digital health innovations offer promising entry points for equity‐oriented PHC transformation. The review concludes that PHC strengthening in Somalia must be pursued as a system‐wide and political priority, anchored in progressive universalism and community partnerships, to deliver sustained improvements in population health while systematically reducing avoidable health disparities.

## Graphical abstract

Primary health care (PHC) strengthening is central to improving population health and advancing health equity in Somalia. Fragmented governance, inadequate financing, workforce maldistribution, and limited access for rural and displaced communities undermine PHC performance. Expanding female community health workers, implementing the Essential Package of Health Services, and leveraging digital health innovations within an equity‐oriented framework can reduce preventable mortality and narrow persistent health disparities.

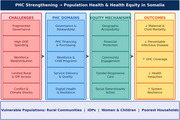

## 1 Introduction

Primary health care (PHC) is widely recognized as the cornerstone of equitable health systems and an essential foundation for achieving universal health coverage (UHC) and sustainable development goals (SDGs) [1]. The global consensus, articulated in the Declarations of Alma‐Ata and Astana, positions PHC as a whole‐of‐society approach that integrates health promotion, disease prevention, treatment, rehabilitation, and palliative care while addressing the broader social determinants of health through multisectoral action and community empowerment [1, 2]. The World Health Organization (WHO) conceptualizes PHC as comprising three core components: integrated health services centered on primary care and essential public health functions, multisectoral policy and action, and empowered people and communities [3]. Robust PHC systems are consistently associated with improved population health outcomes, reduced health inequities, and greater system resilience attributes, which are particularly critical in fragile and conflict‐affected settings [4, 5].

Somalia exemplifies the profound challenges faced by countries that are emerging from protracted conflicts and recurrent humanitarian crises [6, 7]. Decades of state fragility have resulted in one of the world's most under‐resourced health systems, with a UHC index among the lowest globally, and a critical shortage of health workers [6, 7]. The country's health infrastructure remains fragmented, with less than one‐third of the population being able to access a functional primary facility within a reasonable distance [6]. These systemic weaknesses are compounded by persistent insecurity, displacement, poverty, and climatic shocks that disproportionately affect rural communities, internally displaced persons (IDPs), women, and children, who experience some of the most severe health inequities in the region [8, 9]. For example, maternal mortality rates remain among the highest worldwide; only 7% of pregnant women receive adequate antenatal care, and IDPs face formidable barriers to essential services owing to legal exclusion, administrative fragmentation, and social marginalization [9, 10].

Despite these daunting challenges, recent policy developments have signaled a paradigm shift in Somalia's approach to health system transformation [6]. The National Transformation Plan (NTP) 2025–2029 emphasizes multi‐stakeholder engagement, evidence‐based priority setting, and accountability mechanisms designed to move beyond fragmented interventions towards a coherent people‐centered system [6]. Central to this agenda is the expansion of PHC coverage, particularly by scaling up female community health workers (FCHWs), rehabilitating rural facilities, strengthening regulatory institutions, leveraging digital innovations, and fostering public–private partnerships (PPPs) [6, 11]. These strategies align with global best practices that highlight the importance of equitable workforce distribution, sustainable financing arrangements prioritizing vulnerable populations, decentralized governance structures responsive to local needs, and integrated service delivery models capable of reaching marginalized groups [1, 2, 12].

The persistent gaps in population health outcomes in Somalia underscore both the urgency and complexity of strengthening PHC [7, 9]. Socioeconomic status and educational attainment remain strong predictors of access to essential services. Geographic disparities are stark, financial risk protection is inadequate for the poorest households, and traditional models often fail to accommodate nomadic or displaced populations [7, 9]. Addressing these inequities requires not only technical reforms but also political commitment to progressive universalism, placing the rights and needs of disadvantaged groups at the center of policy design and resource allocation [2, 7].

This narrative review aims to critically synthesize evidence on how strengthening PHC can improve population health and reduce health inequities in Somalia, using a systems‐based perspective to examine key health system domains, including governance, financing, workforce, service delivery, quality, resilience, and action on the social determinants of health in a fragile and conflict‐affected context.

## 2 Methods

### 2.1 Study Design

This study adopted a **narrative review design** [13] to systematically synthesize and interpret existing evidence on the role of PHC strengthening in improving population health and advancing health equity in Somalia. A narrative approach was chosen to accommodate diverse sources of evidence, including empirical studies, policy documents, and global health frameworks, which are particularly relevant in fragile and conflict‐affected settings, where standardized empirical data are limited.

### 2.2 Literature Sources

Relevant literature was identified from major academic databases, including PubMed, Google Scholar, Scopus, and Web of Science. In addition, gray literature was consulted from key global and regional institutions, notably the WHO and the World Bank, to capture policy‐oriented and context‐specific evidence not always represented in peer‐reviewed journals. The search strategy combined keywords related to “primary health care,” “health system strengthening,” “health equity,” “population health,” and “Somalia” or “fragile and conflict‐affected settings,” using Boolean operators where appropriate.

### 2.3 Evidence Selection

Sources were selected on the basis of their relevance to PHC, health system strengthening, population health, and health equity in Somalia or comparable fragile contexts. Priority was given to peer‐reviewed articles, high‐quality reviews, and authoritative policy reports, with an emphasis on literature published within the past decade, along with seminal global declarations and frameworks that underpin contemporary PHC policy. Studies were included if they (1) addressed PHC or health system strengthening in relation to population health or equity, (2) focused on Somalia or comparable low‐income and fragile settings, and (3) provided empirical, conceptual, or policy‐relevant insights. Studies were excluded if they lacked relevance to PHC or equity, were opinion‐based without analytical contribution, or did not provide sufficient methodological or contextual detail. The assessment of “high‐quality” evidence was based on factors such as publication in peer‐reviewed journals, methodological clarity, relevance to the research question, and institutional credibility for gray literature (e.g., WHO and World Bank).

### 2.4 Narrative Synthesis

The included evidence was narratively synthesized to describe key themes related to PHC system domains, including governance, financing, workforce, service delivery, quality and resilience, and community engagement, while maintaining a consistent focus on equity implications for rural populations, IDPs, women, and children. The synthesis aimed to integrate global evidence with Somalia‐specific insights to inform policy‐relevant discussions, rather than to quantitatively analyze or compare study outcomes.

Themes were identified through an iterative deductive–inductive process, whereby established PHC system domains informed the initial analytical framework, and recurring patterns emerging from the literature were used to refine and contextualize themes relevant to Somalia's health system and equity challenges. This approach ensured alignment with the study objectives and coherence between the conceptual framework and the results presented.

## 3 Results

**FIGURE 1:**
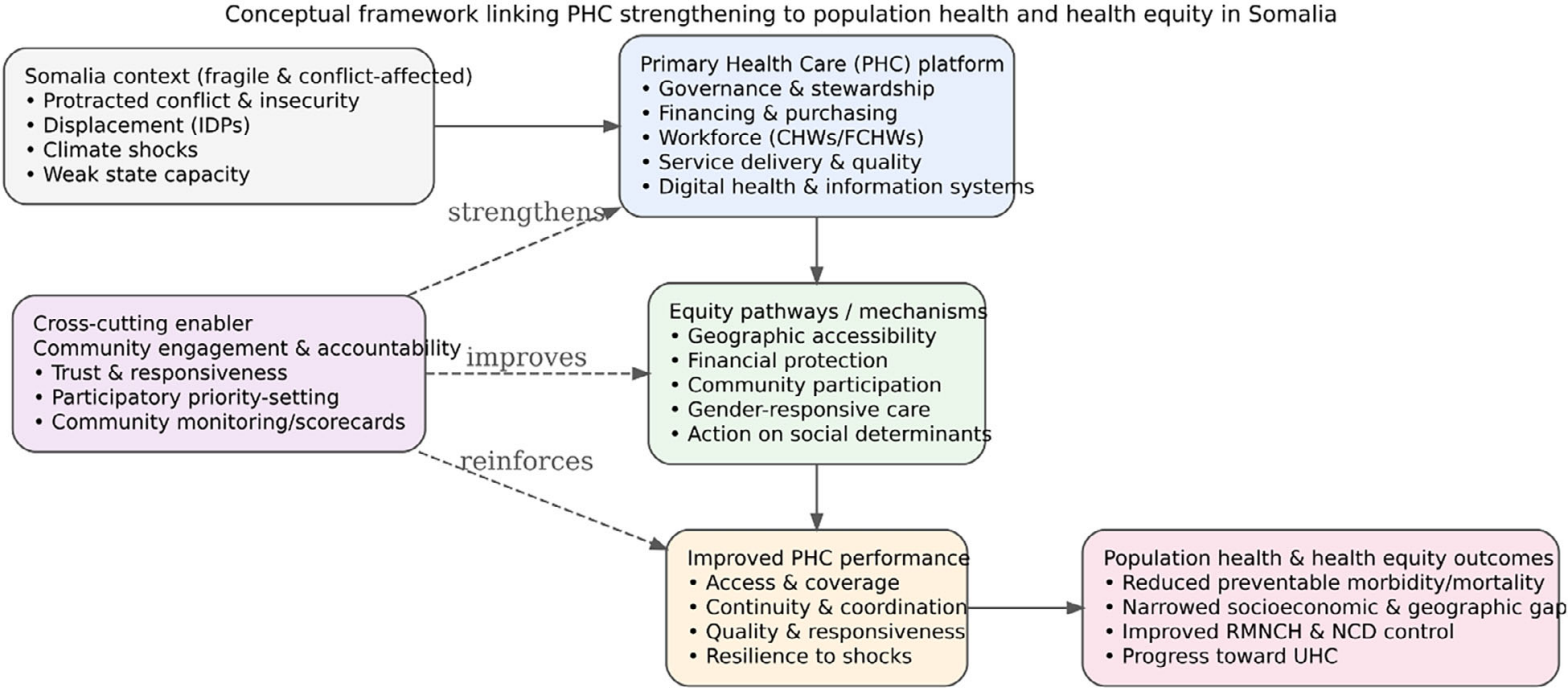
Conceptual framework linking primary health care strengthening to population health and health equity in Somalia. CHW, community health worker; FCHWs, female community health workers; IDPs, internally displaced persons; NCD, noncommunicable disease; RMNCH, reproductive, maternal, newborn, and child health; UHC, universal health coverage.

Figure 1 illustrates a conceptual framework in which strengthening PHC functions as a system‐wide pathway for improving population health and advancing health equity in Somalia's fragile and conflict‐affected context. The figure shows how the PHC platform comprises governance and stewardship, financing and purchasing, workforce development (including community health workers [CHWs]), service delivery and quality, and digital health and information systems operating within an environment shaped by protracted conflict, displacement, climate shocks, and weak state capacity. Investments in these PHC components act through equity‐oriented mechanisms, including improved geographic accessibility, financial protection, community participation, gender‐responsive care, and action on the social determinants of health, to enhance PHC performance in terms of access, continuity, quality, and resilience to shocks. Community engagement and accountability are depicted as crosscutting enablers that strengthen PHC functions, improve equity pathways, and reinforce system performance by fostering trust, responsiveness, and social cohesion. Through these interconnected processes, Figure 1 demonstrates how strengthened PHC can reduce preventable morbidity and mortality; narrow socioeconomic and geographic health gaps; improve maternal, newborn, child, and noncommunicable disease (NCD) outcomes; and support sustained progress towards UHC in Somalia.

### 3.1 Overview of Somalia's Health System and PHC Architecture

Somalia's contemporary health system is characterized by a fragile mix of federal stewardship, state‐level variation, and heavy reliance on non‐state and external actors [14, 15]. The Federal Ministry of Health nominally sets national policy and UHC ambitions, but the implementation authority and resource control are dispersed across federal member states, each with heterogeneous capacities and regulatory arrangements [6, 15]. This fragmented federal–state dynamic constrains the development of a coherent PHC architecture capable of advancing equitable population health and progressing towards UHC, particularly in rural, insecure, and displacement‐affected areas [7, 15]. At the service delivery level, PHC is organized through a tiered network of community‐level posts and primary facilities, including health posts, primary health units, and mother‐and‐child health (MCH) centers, often complemented by FCHWs and mobile outreach [6, 16]. Coverage remains highly uneven; recent analyses suggest that less than one‐third of Somalis can access a functional primary facility within a reasonable distance, with deficits most acute in rural and nomadic communities and in informal settlements hosting IDPs [7, 17]. Where they function, PHC platforms are central to reproductive, maternal, newborn, and child health (RMNCH), immunization, and basic nutrition interventions that underpin reductions in under‐five mortality and child malnutrition; however, these gains have been inequitable, with persistent income‐ and education‐related gaps in skilled birth attendance, contraceptive access, and care‐seeking for sick children [16, 18]. Somalia's health financing landscape further entrenches inequity [7, 14]. The country scores among the lowest globally on UHC indices, with service coverage and financial protection constrained by chronic underfunding, shortages of health workers, and weak infrastructure [6, 7]. Out‐of‐pocket payments remain a dominant financing modality, exposing the poorest households to catastrophic expenditures and deterring care‐seeking, thereby generating substantial unmet needs [7, 14]. Donor‐funded programs frequently implemented through international and national NGOs play a central role in maintaining PHC delivery, particularly in conflict‐affected and hard‑to‐reach areas [14, 16]. Although such interventions can strengthen selected health‐system “building blocks” (e.g., workforce training, supply chains, and community engagement), they are often projectized, short‑term, and weakly integrated into national PHC plans and pooling arrangements, limiting their contribution to sustainable, equitable coverage [2, 6]. Non‐state actors, including private for‐profit providers and faith‑based or charitable facilities, occupy a large share of Somalia's PHC landscape, especially in urban centers [14, 15]. However, regulatory capacity has historically been minimal, leading to uneven quality, variable adherence to national norms, and user fees that systematically disadvantage low‐income, rural, and displaced populations [15, 19]. Recent governance reforms, such as the establishment of the National Health Professionals Council (NHPC), aim to address workforce quality and distribution, yet health worker density remains far below WHO thresholds and heavily skewed towards urban settings and male providers, undermining culturally acceptable and gender‑responsive PHC for women and girls [6, 19]. Structurally, Somalia's PHC system is thus marked by geographic and socioeconomic inequities, weak public financing, and fragmented governance, which collectively impede its potential to drive population health improvement and health equity [7, 15]. Evidence from fragile and conflict‐affected African settings indicates that without deliberate pro‑equity PHC reforms spanning financing, regulation of mixed providers, community‐based outreach, and gender‐responsive service design, UHC risks becoming a “mirage” for conflict‐affected, rural, and displaced populations [2, 20]. For Somalia, re‑centering PHC within a unified, equity‑oriented federal–state framework, backed by progressive financing and accountable PPPs, is therefore not merely a technical priority, but a prerequisite for realizing measurable and fairly distributed gains in health [6, 7].

Table 1 provides a concise synthesis of the PHC system domains examined in this review by mapping the key structural barriers in Somalia to the corresponding equity‐focused reform priorities. This highlights how fragmentation in governance, heavy reliance on out‐of‐pocket and donor financing, workforce maldistribution, gaps in service delivery for rural and displaced populations, and weaknesses in quality and resilience collectively undermine equitable access to care. By aligning each challenge with targeted reform actions, such as unified stewardship, pooled and progressive financing, scale‐up of FCHWs, outreach‐oriented service models, and continuous quality improvement, the table operationalizes the conceptual framework and clarifies how system‐level PHC reforms can be translated into practical, equity‐oriented policy priorities in Somalia.

**TABLE 1:** Summary of primary health care (PHC) system domains, key barriers, and equity‐focused reform priorities in Somalia.

PHC domain	Key barriers identified	Equity‐oriented reform priorities
Governance	Fragmented federal–state authority; weak regulation	Unified stewardship; equity indicators; accountability
Financing	High out‐of‐pocket spending; donor fragmentation	Pooled PHC financing; progressive universalism
Workforce	Urban concentration; gender imbalance	Scale‐up FCHWs; rural retention incentives
Service delivery	Limited rural/IDP coverage; weak referrals	Outreach, mobile PHC, integrated packages
Quality and resilience	Stock‐outs; weak supervision	CQI, digital monitoring, shock preparedness

Abbreviations: FCHWs, female community health workers; IDP, internally displaced person.

Building on this system‐level analysis, the subsequent section examines the population health burden and PHC‐relevant health needs in Somalia, demonstrating how structural weaknesses in governance, financing, workforce, and service delivery translate into observable patterns of disease, unmet needs, and inequitable health outcomes.

### 3.2 Population Health Burden and PHC‐Relevant Health Needs

Somalia's contemporary disease burden is characterized by a convergence of preventable communicable diseases, severe maternal and child health challenges, undernutrition, and a rising tide of NCDs, all of which unfold within a protracted conflict and displacement context [18, 21]. These conditions are largely amenable to PHC, yet current PHC capacity is inadequate, contributing to persistent avoidable morbidity, mortality, and entrenched health inequities [22]. Recurrent outbreaks of cholera, measles, and polio, driven by routine immunization completeness estimated at roughly one‐fifth and weak water, sanitation, and hygiene (WASH) systems, exemplify the consequences of fragile PHC and surveillance platforms in Somalia [23, 24]. A recent narrative review linked these outbreaks to a health workforce density below 0.4 doctors, nurses, and midwives per 10,000 population, poorly equipped facilities, and patchy epidemic intelligence [23, 25]. Community‐based mortality surveillance among IDPs in the Banadir further demonstrates that severe malnutrition, respiratory infections, diarrheal diseases, and measles dominate mortality, particularly among children under five, underscoring the failure to deliver basic PHC interventions such as vaccination, integrated management of childhood illness, and nutrition support at scale [24, 26]. These patterns are consistent with global evidence that women and children living near armed conflict suffer substantial excess mortality from nonviolent causes, largely mediated through infectious diseases, malnutrition, and disrupted reproductive, maternal, and newborn care [27, 28]. Maternal and child health outcomes are particularly sensitive to PHC in fragile settings [28, 29]. Multicountry conflict analyses show that antenatal care, basic and comprehensive emergency obstetric and newborn care, immunization, and treatment of common childhood illnesses are the core women's and children's health interventions prioritized where PHC is functional, yet coverage remains incomplete, and reproductive, adolescent, and stillbirth prevention interventions are often absent [22, 28]. In Somalia, stakeholder consultations for the NTP identify high maternal and under‐five mortality, limited rural coverage, and infrastructure deficits as central PHC‐relevant gaps and emphasize that expanding FCHWs and rehabilitating primary‐level facilities are essential to reach remote and marginalized communities, including pastoralists and IDPs [23, 30]. Weak PHC further reinforces spatial, gender, and socioeconomic gradients in survival and service use [18, 22]. Somalia's malnutrition burden, particularly in IDP camps affected by drought and conflict, has become a major driver of mortality, with verbal autopsies in Banadir attributing a large share of deaths, especially among young children, to severe malnutrition often combined with respiratory and diarrheal infections [24, 26]. Climate‐sensitive shocks, including recurrent droughts and floods, exacerbate food insecurity and unsafe water, expanding the pool of susceptible individuals and precipitating infectious disease surges where PHC‐based nutrition, WASH, and outbreak‐preparedness functions are weak [23, 31]. Global reviews of conflict and infectious diseases confirm that displacement, infrastructure destruction, interrupted vaccination, and reduced access to care are key pathways through which conflict multiplies epidemic risk, all of which are highly salient in Somalia [27, 31]. Simultaneously, Somalia is undergoing an epidemiological transition with rising NCDs and mental health needs that are poorly addressed by the current PHC configurations [21, 32]. Global frameworks emphasize that PHC is the locus for NCD prevention and life‐course management of chronic conditions, including hypertension, diabetes, and chronic respiratory disease [28, 32]. However, Somalia's extremely low UHC index and limited PHC infrastructure mean that early detection, continuity of care, and financial protection for NCDs are minimal, pushing patients towards catastrophic out‐of‐pocket expenditures or foregone care, especially among the poorest and least educated groups [18, 22]. Prolonged conflict, displacement, and exposure to violence also generate high burdens of common mental disorders and trauma‐related conditions; however, mental health is rarely integrated into PHC in fragile settings, contributing to unaddressed psychosocial distress among women, adolescents, and displaced populations [28, 33]. Evidence from Somalia and comparable contexts indicates that IDPs experience systematically worse health outcomes than other conflict‐affected populations due to reduced access to services, with camp‐based studies documenting a high prevalence of acute, preventable conditions such as diarrhea, fever, respiratory symptoms, and significant access barriers related to distance, transport, stock‑outs, and cost [17, 26]. In Somalia, less than one‐third of the population is estimated to live within a reasonable distance from a functional primary facility, with rural, nomadic, and displaced communities most affected [23, 30]. This spatial exclusion interacts with poverty, gender norms, and disability to deepen inequities in service utilization and health outcomes [18, 22]. At the system level, Somalia's UHC index remains among the lowest globally, with pronounced pro‐rich inequities in access to basic services and heightened risk of catastrophic health expenditure among the poorest households [18, 22]. Global PHC and resilience analyses show that when PHC is underfunded, fragmented, and of poor quality, people either bypass primary facilities or do not seek care at all, perpetuating late presentations, preventable deaths, and financial hardship [28, 32]. In Somalia, the combination of low public spending on PHC, donor‐dependent vertical programs, and weak governance under federalism constrains the capacity of PHC to address the main population health priorities and to buffer the impacts of conflict and climate shocks [23, 30]. Collectively, these patterns demonstrate that Somalia's current health burden is highly PHC‐relevant and that deficiencies in PHC financing, service delivery, workforce, and quality disproportionately harm vulnerable groups of rural populations, IDPs, women, children, and persons with disabilities, thereby entrenching inequities [18, 28]. Strengthening people‑centered, equity‑oriented PHC through expanded geographic coverage, integrated infectious disease and NCD management, maternal and child health services, mental health care, and climate‑sensitive public health functions is, therefore, indispensable to improving population health and advancing health equity in Somalia and other fragile, conflict‐affected states [23, 28].

### 3.3 Health Inequities and Vulnerable Populations

Health inequities in Somalia are shaped by intersecting determinants of geography, gender, displacement, and socioeconomic position, which together structure highly unequal access to PHC and, consequently, unequal risks of morbidity and mortality [9, 18]. Somalia's very low UHC index and pro‑rich gradients in service use and financial risk protection indicate that PHC does not function as an equity‑enhancing platform, but instead mirrors and sometimes amplifies broader social stratification [2, 18]. Therefore, addressing these inequities through deliberate PHC redesign is central to improving population health and advancing health equity in this fragile and conflict‐affected setting [2, 34]. The geographical and livelihood‐based disparities are profound [9, 35]. Analysis of the 2020 Somalia Demographic and Health Survey showed that only around 7% of pregnant women achieved the minimum of four antenatal care visits, with nomadic women having 90% lower odds and rural women 70% lower odds of adequate antenatal care compared to urban residents [9]. Similarly, the UHC analysis finds that service coverage and financial protection remain systematically worse for poorer, less educated households and in areas with weaker infrastructure, reflecting an inequitable distribution of PHC inputs [2, 18]. These patterns indicate that current PHC delivery models remain overly urban‑centered and facility‑based, failing to reliably reach rural, pastoralist, and hard‑to‑reach communities, despite emerging evidence that FCHW programs can extend essential maternal and child health services to remote populations [16, 35]. The displacement status is a major axis of exclusion [36, 37]. Somalia hosts a large number of IDPs who face overcrowding, insecure tenures, and precarious livelihoods [16, 36]. IDP‐focused studies in Mogadishu and Baidoa have reported significant barriers to care, including transport costs, distance to facilities, long waiting times, medicine stock‑outs, and facility closures, all of which constrain the timely use of PHC services [17, 38]. Only one‐quarter of women in IDP camps in Benadir delivered at health facilities, with home births driven by financial constraints, distant or closed facilities, lack of transportation, and fear of procedures [38]. For childhood vaccination, IDP caregivers describe multilevel barriers spanning fear and misinformation, domestic power imbalances, eviction threats, stock‑outs, and confusing eligibility rules, leading to delayed or missed immunizations [36, 37]. These findings are consistent with broader evidence that IDPs in conflict settings exhibit systematically worse health outcomes and lower service coverage than other conflict‐affected groups because of their reduced access to PHC [37]. Therefore, in Somalia, PHC is not yet structured as a migration‑aware, inclusive system; displaced populations remain peripheral to planning and entitlement regimes [39]. Gender and age further intersect with socioeconomic positions to shape inequities [18]. The evaluation of maternal and newborn health equity trends from 2006 to 2019 shows that although reductions in child stunting and wasting differences have contributed to narrowing the under‐five mortality gaps, inequalities in contraceptive access, skilled birth attendance, and care‑seeking for sick children have widened to the disadvantages of poorer women and their children [18]. These inequities are reinforced by a critically low and male‐dominated health workforce (0.92 health workers per 1000 population; 78% male), which undermines women's ability to express health concerns and obtain respectful, gender‑sensitive PHC [16, 18]. Qualitative work in IDP settings highlights women's low decision‐making power, reliance on traditional birth attendants, and pervasive financial dependency as structural barriers to using maternal and child health services even when nominally free facilities exist [38]. Children's nutritional status and survival are closely linked to mothers’ education; better‑educated mothers have improved child nutrition and lower under‐five mortality, underscoring the role of broader social determinants and the need for PHC that is explicitly pro‐poor, sex‐responsive, and integrated with educational and social protection policies [18, 40]. Socioeconomic gradients have cut across these domains [18, 40]. UHC equity analysis shows that higher household income and education are strongly associated with better access to essential services and a lower risk of catastrophic health expenditure, revealing that out‐of‐pocket financing remains a key barrier to PHC for the poorest groups [2, 18]. Studies of maternal and child health and IDP health‑seeking behavior similarly document that the cost of care, medicines, and transport are central reasons for delayed or forgone PHC [17, 38]. From a global PHC financing perspective, such reliance on out‐of‐pocket payments and fragmented purchasing arrangements predictably entrenchs inequity, which requires that pooled public funds first secure free, high‐quality PHC for the poorest and most vulnerable [2, 34]. Taken together, these patterns show that the current PHC arrangements in Somalia insufficiently protect those with the greatest need for rural communities, IDPs, women, children, and the poorest households, thereby reproducing avoidable differences in exposure to risk, access to timely care, and health outcomes [9, 17, 35]. Re‑orienting PHC towards geographically inclusive delivery models (including community and mobile approaches), gender‑balanced and community‑embedded workforces, and equity‑focused financing is, therefore, not only a technical imperative but also an ethical and political one [2, 16, 35]. Only an explicitly equity‑driven PHC strategy can shift Somalia from a system that manages inequity to one that systematically reduces it, thereby improving overall population health while narrowing the unjust gaps in survival and well‐being [2, 18].

### 3.4 Barriers to Effective PHC Delivery

PHC in Somalia is constrained by interlocking governance, financing, workforce, service delivery, and quality‐of‐care barriers that systematically undermine population health gains and entrench inequities, particularly among rural communities, IDPs, women, and children [7, 15]. At the governance level, fragmentation is the defining obstacle [14, 15]. Authority is dispersed across federal and state ministries, de facto authorities, a large humanitarian–NGO community, and an expanding private sector with weak regulatory capacity and limited accountability mechanisms [6, 16]. The NHPC remains dependent on registration fees, undermining sustainable stewardship over standards and professional regulation [6, 19]. Humanitarian health responses for women and children are shaped as much by donors’ priorities and short funding cycles as per population needs, perpetuating a projected PHC landscape rather than an integrated public system [16, 18]. This fragmentation hampers the coherent implementation of the Essential Package of Health Services (EPHS) and weakens the ability to plan for equitable PHC expansion, particularly in conflict‐affected and hard‐to‐reach areas [15, 18]. Health financing is both insufficient and structurally misaligned with PHC and equity goals [14, 18]. Somalia's UHC index remains among the lowest globally, reflecting underfunding, heavy out‐of‐pocket payments, and a high risk of catastrophic expenditure among the poorest households [7, 18]. Donor dependence and the bifurcation between humanitarian and development aid lead to parallel funding streams that privilege vertical projects and emergency responses over sustained primary care, continuity of services, and financial protection [14, 16]. User fees in both public and private facilities constitute a major barrier to timely access to emergency and essential services, disproportionately affecting the poorest, rural areas, and IDP camps [7, 41]. These financing patterns reinforce socioeconomic gradients in service use and exacerbate the exclusion of marginalized groups from essential PHC [7, 18]. Severe workforce shortages and maldistribution undermine the availability and quality of PHC [6, 19]. Somalia's clinician density is critically low, and staff are concentrated in major cities, leaving rural districts, pastoralist communities, and many IDP settlements with minimal or no skilled providers [6, 15]. Health workers often juggle multiple jobs across NGOs and private facilities, reducing their availability to public PHC and weakening the continuity of care [16, 17]. Insecurity, poor working conditions, and a lack of incentives further deter deployment and retention in underserved areas [6, 38]. From a quality perspective, inadequate training and weak supervision contribute to disrespectful care, limited competence, and poor responsiveness, particularly in maternal and reproductive health services for both displaced and poor women [6, 17]. Service delivery is characterized by gaps in geographic coverage, weak referral systems, and dependence on humanitarian modalities [15, 16]. Less than one‐third of the population is estimated to live within a reasonable distance from a functional primary facility, with infrastructure deficits, especially acute in rural and conflict‐affected districts [6, 42]. Access barriers are amplified by long travel distances, unreliable transport, closures, or reduced operating hours linked to insecurity [17, 38]. Referral pathways between community, primary, and higher level care are often informal or nonfunctional, with IDP populations reporting a lack of clear referral mechanisms and episodic closure of facilities [17, 38]. For women, children, and adolescents in conflict settings, essential PHC‐linked services (e.g., comprehensive reproductive health, adolescent services, and stillbirth prevention) are frequently absent or only partially delivered despite being highly prioritized in global guidance [16, 19]. His discontinuity in PHC undermines prevention, early treatment, and chronic disease management, contributing to high maternal, neonatal, and under‐five mortality rates and rising NCD burdens [9, 18]. Quality‐of‐care deficits at the PHC level are both structural and process‐related [16, 43]. System‐wide assessments highlight shortages of drugs, basic supplies, and functional equipment along with inadequate readiness for emergency and critical care at first‐level facilities, particularly in the public sector [41, 44]. Providers report being overburdened and under‐supported, which, combined with limited training in respectful and person‐centered care, results in neglect, verbal abuse, and poor communication, deterring service use among vulnerable groups [17, 38]. In fragile and conflict‐affected settings, similar patterns of non‐responsive, inequitable care, and weak surveillance constrain health system resilience and the realization of UHC [7, 43]. For Somalia, these quality failures mean that even when services are nominally available, they may not translate into effective coverage or improved health outcomes for the poorest and most marginalized [7, 18]. Conflict, climate change, and recurrent humanitarian emergencies intersect these systemic weaknesses, further disrupting PHC and deepening inequities [16, 45]. Protracted insecurity restricts the mobility of patients and staff, leads to attacks on health facilities, and interrupts supply chain mechanisms well‐documented in other conflict settings and echoed in Somalia's experience [9, 16]. Droughts, floods, and displacement drive periodic surges in malnutrition and infectious diseases, overburdening already fragile PHC facilities and diverting resources towards short‐term emergency campaigns rather than sustained, integrated primary care [16, 45]. Public health emergencies in weak systems, such as Somalia, are associated with service interruptions, widening inequities, and near‐collapse under shock, particularly at the first point of contact [42, 46]. These dynamics disproportionately harm rural communities, IDPs, women, and children whose access to PHC is already the most tenuous, thereby entrenching spatial, gender, and socioeconomic health inequities [7, 38]. Taken together, these barriers indicate that without deliberate system‐wide strengthening of PHC governance, financing, workforce, service delivery, and quality explicitly designed for resilience to conflict and climate shocks, Somalia will struggle to translate primary care expansion into meaningful improvements in population health and health equity [6, 7].

### 3.5 Opportunities and Enablers for Strengthening PHC

#### 3.5.1 Policy and Strategic Frameworks (EPHS and NTP)

Despite its profound fragility, recent reforms and innovations provide a substantive opportunity structure for advancing a more equitable, PHC‐oriented health system in Somalia [6, 47]. The revision of the EPHS in 2020 through an inclusive and technically grounded process is a central enabler [47, 48]. By prioritizing high‐impact, cost‐effective interventions and sequencing their roll‑out across levels of care, the EPHS explicitly aims to address the major causes of mortality and progressively extend coverage to all populations, including those in security‑compromised and remote areas [47, 49]. This progressive realization approach, aligned with UHC principles, creates a policy lever for directing scarce resources towards PHC services that most directly reduces avoidable maternal and child deaths and mitigates inequities between urban and rural, wealthy, and poor households [47, 50]. If fully financed and implemented, it can reorient the system from ad hoc humanitarian provision to a more standardized rights‐based package of primary care [47, 49].

#### 3.5.2 Community Health Workforce Expansion

CHW and, specifically, FCHW models constitute another critical opportunity for equity‐promoting PHC expansion [6, 51]. The National Female Community Health Worker and Marwo Caafimaad programs deploy locally recruited women to deliver home‐based services for antenatal care promotion, immunization follow‑up, management of common childhood illnesses, and basic reproductive health, particularly in rural and nomadic communities that face the most severe physical and financial barriers to facility‐based care [51, 52]. Evidence indicates that these cadres improve maternal and child health coverage, build trust through gender‐ and culturally congruent care, and extend the government presence in underserved settings [51, 52]. Stakeholders in Somalia's NTP (2025–2029) similarly identified the scale‐up of female CHWs and rehabilitation of rural health posts as the fastest route to increasing PHC coverage beyond the current low levels [6, 51]. Optimizing and integrating CHW programs within the EPHS offers a practical pathway to reaching IDPs and marginalized rural clans with preventive and promotive care that directly narrows health inequities [51, 53].

#### 3.5.3 Digital Health and Service Delivery Innovations

Digital health innovations are an emergent but powerful enabler of PHC strengthening and governance reforms [42, 52]. The rollout of DHIS2‐based systems and SMS/voice complaint mechanisms enhances routine data availability, supports quasi‐experimental tracking of maternal and child health indicators, and creates real‐time feedback loops between communities and authorities [52, 54]. Telemedicine platforms and mobile applications are increasingly being used to bridge geographic gaps in access and to support immunization and clinical decision‐making in settings with limited specialist presence [6, 42]. When embedded at the PHC level, such tools can reduce missed opportunities for care, improve accountability for service delivery, and enhance resilience during insecurity or climate shocks by maintaining virtual links between frontline providers and referral centers [52, 54]. Nevertheless, digital inequities (connectivity, literacy, and gender gaps) must be explicitly addressed to avoid reinforcing the existing disparities [42, 55].

#### 3.5.4 Governance, Financing, and System Integration

Somalia's evolving governance and financing architecture also create enabling conditions [6, 50]. The EPHS revision, multi‐stakeholder NTP 2025–2029 consultations, and growing federal–state coordination mechanisms demonstrate an emerging platform for more coherent PHC policy, regulation, and performance management [6, 47]. Proposals for PHC coverage scorecards, joint inspection audits, and performance‐linked PPP contracts offer concrete instruments to align fragmented public, private, and humanitarian providers around equity‐oriented PHC targets [6, 56]. In parallel, efforts to integrate humanitarian RMNCAH and nutrition services with government‐led PHC planning framed within the humanitarian–development–peace nexus provide an opportunity to convert short‐term emergency inputs into long‐term district‐level PHC capacity, supply chain strengthening, and workforce development [49, 57]. Global evidence on PHC reforms underscores that such integrated, community‑engaged, and digitally enabled PHC platforms are the most cost‐effective route to UHC, particularly for marginalized populations in low‐ and middle‐income and conflict‐affected settings [58, 59]. Harnessing these opportunities with explicit pro‑poor, gender‑transformative, and rural‑focused strategies is therefore pivotal if Somalia translates PHC strengthening into tangible gains in population health and equity [6, 50].

## 4 Discussion

### 4.1 Strategic Pathways for PHC Strengthening

Advancing population health and health equity in Somalia requires deliberate reorientation of the health system around a resilient, people‐centered PHC platform [2, 60]. Evidence from low‐ and middle‐income countries (LMICs) and fragile and conflict‐affected settings shows that well‐governed, adequately financed, community‐embedded PHC systems improve service coverage, reduce preventable mortality, and narrow socioeconomic and geographic inequities, particularly when reforms explicitly target disadvantaged populations and the social determinants of health [61, 62]. Translating this evidence to Somalia's context of protracted fragility, urban–rural disparities, and large internally displaced populations demands context‐specific but globally informed strategic pathways [1, 63]. These strategic pathways are interdependent and collectively aim to address the structural constraints identified in Somalia's PHC system while advancing equitable population health outcomes.

#### 4.1.1 Strengthening PHC Governance

First, strengthening PHC governance is fundamental to equity‐oriented reform [64, 65]. Comparative reviews highlight that governance functions, such as strategic policy formulation, regulation, decentralization with clear accountability, generation and use of evidence, and multisectoral coordination, are central to PHC‐oriented progress towards UHC [64, 66]. In fragile and conflict‐affected states, coordinated, integrated responses tailored to crisis stages and local contexts, with strong community involvement, are critical to overcoming fragmentation and building trust [63, 67]. In Somalia, the establishment of the NHPC and the participatory NTP 2025–2029 created an institutional entry point to align federal and state actors, regulate rapidly proliferating training institutions, and standardize PHC quality [6]. Embedding equity metrics, such as rural PHC coverage, service use among IDPs, and gender‐responsive indicators within routine monitoring and public scorecards, can link governance reforms directly to reductions in avoidable maternal and under‐five deaths in rural and displacement‐affected communities [64, 66]. However, governance reforms must be accompanied by investments in data systems and regulatory capacity, for which the current evidence in Somalia remains limited [15, 68].

#### 4.1.2 Reforming PHC Financing

Second, PHC financing must be redesigned to reduce catastrophic payments and channel resources towards the underserved population [2, 61]. Across LMICs, high out‐of‐pocket expenditures and underfunded PHC packages undermine equity, whereas equity‑informed financing models and increased public spending on PHC are associated with improved access, financial protection, and health outcomes [34]. Analyses of PHC systems recommend that governments increase primary care spending by at least 1% of the gross domestic product and develop equity‐enhancing schemes that prioritize disadvantaged locations and populations [1, 69]. For Somalia, where domestic fiscal space is constrained and external aid is fragmented, a phased strategy is required: progressively raise domestic allocations for an explicit PHC benefits package, pool donor funds within a PHC‐oriented UHC framework, and use targeted purchasing and performance‐linked payments to incentivize rural facilities, outreach to IDPs, and provision of essential maternal, child, and communicable disease services [15, 34]. Carefully regulated PPPs in PHC, supported by clear contractual frameworks and performance review mechanisms, can expand access in underserved areas if they are aligned with public priorities and monitored for equity impacts [5, 70]. Without such financing reforms, PHC expansion risks entrenching the existing urban and wealth gradients in service use [71, 72].

#### 4.1.3 Building an Equitable Health Workforce

Third, building a competent and equitably distributed PHC workforce is essential for realizing population‐level gains [63, 66]. Reviews of health systems in fragile states underscore the importance of policies encouraging the retention and return of health workers, local capacity building, and supportive supervision for community and mid‑level cadres [63, 66]. CHWs and other nonphysician health workers are consistently associated with expanded coverage and improved quality of care when they are well‐integrated, remunerated, supervised, and supplied [66, 69]. In Somalia, the extremely low density of clinicians and marked urban–rural maldistribution require a dual strategy: strengthening the regulation and accreditation of rapidly growing health professions education to ensure quality, while scaling up and formalizing FCHWs and other CHWs linked to PHC teams in rural, nomadic, and IDP communities [6, 68]. Global frameworks for measuring community health workforce performance emphasize incentives, supervision, data use, service quality, and community trust as core dimensions of program management [66, 69]. In Somalia, such metrics can guide investments that improve the continuity of antenatal care, immunization, and management of childhood illness in hard‑to‑reach populations, translating directly into reduced mortality and narrower equity gaps [62, 73].

#### 4.1.4 Integrating Priority Health Services

Fourth, integrating priority services within PHC, including NCD care and neglected tropical diseases such as visceral leishmaniasis, is necessary to address Somalia's evolving disease burden without deepening inequities [1, 2]. Evidence from LMICs in Asia‐Pacific and global rapid reviews indicates that integrated PHC combining preventive, promotive, and curative services and linking community and facility levels improves coverage, efficiency, and responsiveness, provided there is compatible policy alignment, adequate health system readiness, and strong leadership [1, 73]. The integration of NCD services into PHC is particularly sensitive to workforce capacity, supply chains, and clear clinical pathways; when these are addressed, integrated models can prevent disease progression and reduce hospitalization costs, benefiting poor households disproportionately [2, 66]. In Somalia, integrating maternal, child, infectious disease, NCD, and NTD services into a coherent PHC package, with referral continuity between community, health posts, and district hospitals, would reduce the current reliance on fragmented vertical programs and tertiary care in urban centers [6, 68]. Such integration must explicitly prioritize rural and conflict‐affected districts where current access to functional PHC facilities is particularly low [6, 68].

#### 4.1.5 Quality Improvement and System Resilience

Quality improvement and resilience‐building within the PHC constitute the fifth strategic pathway [63, 67]. Global evidence shows that investments in PHC improve not only access but also system accountability, responsiveness, and resilience to shocks such as COVID‑19 when accompanied by quality improvement mechanisms, managerial capacity, and robust supply systems [61, 67]. In Southeast Asia, pandemic‐catalyzed workforce mapping, task‐shifting, and digital platforms within PHC have maintained essential services and enhanced surge capacity [1, 67]. For Somalia, institutionalizing continuous quality improvement through standard treatment guidelines, supportive supervision, facility audits linked to public reporting, and the use of PHC performance indicators can raise the quality of care for women, children, and people with chronic conditions in both public and contracted private facilities [6, 64]. Strengthening supply chain management for essential medicines and diagnostics at the PHC level will further reduce inequitable treatment gaps, particularly in rural and displaced populations [66, 72]. Nonetheless, empirical evidence on feasible quality improvement models in Somalia is scarce, underscoring the need for implementation research embedded in PHC reforms [61, 64].

#### 4.1.6 Community Engagement and People‐Centered PHC

Finally, community engagement and people‐centered PHC are critical levers for equitable impacts [1, 69]. Scoping reviews demonstrate that community health programs and community‐controlled governance structures enhance access, modify social determinants of health, and reduce care costs, especially for marginalized groups [61, 64]. Community engagement and social participation underpin trust, service uptake, and accountability and are increasingly recognized as the core of PHC and UHC strategies [67, 73]. In Somalia, the scaling up of FCHWs, neighborhood health committees, and partnerships with religious and traditional leaders has been identified by stakeholders as pivotal to extending PHC to remote settlements and addressing gender and cultural barriers to care [6]. Embedding mechanisms for community voice in PHC planning, such as community scorecards, participatory priority setting, and feedback platforms, can ensure that services respond to the needs of rural households, IDPs, and other vulnerable groups, thereby aligning PHC investments with the goal of “leaving no one behind” [64, 69]. When combined with multisectoral action on water, nutrition, education, and livelihoods, such people‐centered PHC approaches can address the upstream determinants of ill health and accelerate progress towards more equitable population health outcomes in Somalia [2, 73].

### 4.2 Implications for Policy, Practice, and Research

Strengthening PHC in Somalia should be framed as a central nation‑ and state‑building priority, rather than a narrow sectoral reform, given that robust PHC systems are strongly associated with improved population health, financial protection, and progress towards UHC in LMICs [1, 2]. This implies consolidating a coherent PHC vision within Somalia's federal and state structures, aligned with the WHO PHC Operational Framework and UHC agenda, and explicitly oriented towards reducing geographic, gender, socioeconomic, and displacement‐related inequities [61, 74]. Equity‑enhancing PHC financing reforms are essential; international evidence shows that high out‐of‐pocket expenditure, fragmented insurance, and underfunded preventive services systematically undermine access for the poorest and conflict‐affected populations, whereas PHC‑oriented public financing and social protection can narrow these gaps [2, 75]. Somalia's recovery strategy should therefore prioritize increasing public allocations to PHC, reducing user fees for essential services, and regulating non‐state providers, drawing on lessons from other fragile and conflict‐affected states where unregulated privatization has deepened inequities and weakened community engagement [75, 76]. At the service delivery level, global and African conflict‐affected experiences show that mixed PHC delivery models combining fixed facilities, mobile clinics, outreach, and community‑based platforms are critical for reaching rural communities, IDPs, women, and children facing insecurity and displacement‐related barriers [77, 78]. Task sharing with CHWs and mid‑level providers, if appropriately trained, supervised, and integrated, has demonstrated the potential to expand essential maternal, child, mental health, and chronic disease services to marginalized populations in fragile settings, but risks poor quality and fragmentation if not embedded in a coherent PHC system [75, 77]. The quality of PHC requires explicit attention, and analyses from multiple fragile states indicate that poor technical and experiential quality, including for the poorest and least educated, drives avoidable mortality, even when facilities exist, underscoring the need for standards, supportive supervision, and accountability mechanisms anchored at the primary care level [61, 79]. Somalia's protracted crisis also demands a resilience‐oriented PHC agenda that strengthens governance, local decision‐making, community participation, and multisectoral action on social determinants, consistent with governance‐centered resilience frameworks and experiences from other fragile contexts [74, 79]. From a practical perspective, investments in district‐level PHC management, digital tools, and integrated public health functions can enable the continuity of essential services during shocks, as observed in countries where PHC platforms have underpinned more effective COVID‑19 and epidemic responses [1, 61]. Research priorities in Somalia should move beyond disease‐specific studies towards implementation and health systems research that examines how PHC reforms are designed, financed, governed, and scaled in a highly fragmented, conflict‐affected environment, addressing the global gap in causal understanding of how governance and system arrangements translate into health and equity gains [61, 74]. There is a particular need for mixed methods, practice‐based, and participatory research on community‑embedded PHC models for displaced and rural populations, innovations in PHC financing and purchasing, digital health at the primary care level, and strategies to integrate NCD, mental health, and reproductive health into a people‑centered PHC platform in fragile settings. Finally, Somalia's experience can contribute to and draw from a growing body of comparative work on PHC and health‐system strengthening in fragile and conflict‐affected states, emphasizing transferable lessons on community‐centered governance, integrated service delivery, and equity‐oriented financing as core pathways to improving population health and health equity.

## 5 Conclusion

This narrative review demonstrates that strengthening PHC is central to improving population health and advancing health equity in Somalia's fragile and conflict‐affected context. The evidence highlights that persistent challenges including fragmented governance, inequitable financing, workforce maldistribution, and limited access for rural and displaced populations continue to undermine PHC performance and reinforce avoidable health disparities. However, emerging opportunities, such as the EPHS, expansion of FCHWs, and digital health innovations, provide a critical foundation for transformation. A coherent, equity‐oriented PHC strategy anchored in progressive universalism, integrated service delivery, and strong community engagement is essential. Prioritizing PHC as a system‐wide and political commitment offers a viable pathway to reduce preventable mortality, strengthen resilience, and achieve sustainable and inclusive health gains in Somalia.

## Author Contributions

Yusuf Abdullahi Hubow conceptualized and coordinated the study and led manuscript drafting. Aniso Mohamed Abdi, Ilyas Abdullahi khalif, and Abas Nor Abdi contributed to development of the conceptual framework and thematic structure. Ahmed Mohamed Omar, Narura Omar Mohamed, Ayan Abdullahi Mohammed, Hibo hassan Mohamed, and Nour Ahmed Dahir conducted literature screening and synthesis. Sharmake Gaiye Bashir and Ahmed Abdinasir Abdulle contributed to drafting and critical revision of key sections. All authors contributed to interpretation of findings, reviewed and approved the final manuscript, and agree to be accountable for all aspects of the work.

## Funding

The authors have nothing to report.

## Ethics Statement

The authors have nothing to report.

## Consent

The authors have nothing to report.

## Conflicts of Interest

The authors declare no conflicts of interest.

## Data Availability

The data that support the findings of this study are available on request from the corresponding author. The data are not publicly available due to privacy or ethical restrictions.

## References

[1] J. C. Alegre , S. Sharma , F. Cleghorn , and C. Avila , “Strengthening Primary Health Care in Low‐ and Middle‐Income Countries: Furthering Structural Changes in the Post‐Pandemic Era,” Frontiers in Public Health 11 (2023): 1270510, 10.3389/fpubh.2023.1270510. 38419816 PMC10899890

[2] K. Hanson , N. Brikci , D. Erlangga , et al., “The Lancet Global Health Commission on Financing Primary Health Care: Putting People at the Centre,” Lancet Global Health 10 (2022): E715–E772, 10.1016/S2214-109X(22)00005-5. 35390342 PMC9005653

[3] World Health Organization & United Nations Children's Fund (UNICEF) , Operational Framework for Primary Health Care: Transforming Vision Into Action (World Health Organization, 2020), https://iris.who.int/handle/10665/337641.

[4] K. C. Stange , W. L. Miller , and R. S. Etz , “The Role of Primary Care in Improving Population Health,” Milbank Quarterly 101 (2023): 795–840, 10.1111/1468-0009.12638. 37096603 PMC10126984

[5] L. Doshmangir , A. Shirjang , M. Bazyar , and V. S. Gordeev , “Primary Health Care Reforms: A Scoping Review,” in Primary Health Care Research & Development, ed. S. Kendall and M. Akman (Cambridge University Press, 2025), 1477–1128, 10.1017/S1463423625000271. PMC12455359 40820273

[6] N. I. Dirie , M. M. Ahmed , Y. B. Abdullahi , et al., “Transforming Health in Post‐Conflict Somalia: Priorities From a Multi‐Stakeholder Roundtable on the 2025–2029 National Plan,” Journal of Healthcare Leadership 17 (2025): 459–468, 10.2147/JHL.S541966. 40964046 PMC12439833

[7] Z. Nikoloski , M. M. Mohamoud , and E. Mossialos , “Universal Health Coverage in Fragile and Conflict‐Affected States: Insights From Somalia,” International Journal for Equity in Health 24 (2025): 125, 10.1186/s12939-025-02486-3. 40336100 PMC12060528

[8] D. J. Chisolm , J. A. Dugan , J. F. Figueroa , et al., “Improving Health Equity Through Health Care Systems Research,” Health Services Research 58 (2023): 289–299, 10.1111/1475-6773.14192. 38015859 PMC10684038

[9] Y. H. Abdi , Y. B. Abdullahi , M. S. Abdi , et al., “Antenatal Care Utilization in Somalia, 2020 Somalia Demographic Health Survey,” Journal of Epidemiology and Global Health 15 (2025): 126, 10.1007/s44197-025-00475-x. 41114755 PMC12537624

[10] M. Jelle , A. J. Seal , and H. Mohamed , “Understanding Multilevel Barriers to Childhood Vaccination Uptake Among Internally Displaced Populations (IDPs) in Mogadishu, Somalia: A Qualitative Study,” BMC Public Health [Electronic Resource] 23 (2023): 2018, 10.1186/S12889-023-16153-1. 37848917 PMC10580585

[11] B. Oyugi , K. Kallander , and A. S. M. Shahabuddin , “Strengthening Primary Health Care Through Implementation Research: Strategies for Reaching Zero‐Dose Children in Low‐ and Middle‐Income Countries' Immunization Programs,” Vaccines 13 (2025): 1040, 10.3390/vaccines13101040. 41150428 PMC12568197

[12] C. C. Butler , R. Mash , N. Gobat , et al., “Democratising Clinical Trials Research to Strengthen Primary Health Care,” Lancet Global Health 13 (2025): E749–E758, 10.1016/S2214-109X(24)00513-8. 40155112 PMC11950428

[13] J. Sukhera , “Narrative Reviews: Flexible, Rigorous, and Practical,” Journal of Graduate Medical Education 14 (2022): 414–417, 10.4300/JGME-D-22-00480.1. 35991099 PMC9380636

[14] A. Y. Ahmed , F. A. Nor , M. Y. Ahmed , et al., “Universal Health Coverage in Somalia: Charting the Path to Equitable Healthcare Financing and Governance,” Health 15 (2023): 1298–1317, 10.4236/health.2023.1511085.

[15] A. S. Said and D. I. Kicha , “Implementing Health System and the New Federalism in Somalia: Challenges and Opportunities,” Frontiers in Public Health 12 (2024): 1205327, 10.3389/FPUBH.2024.1205327. 38362207 PMC10867962

[16] Z. Ahmed , A. Ataullahjan , M. F. Gaffey , et al., “Understanding the Factors Affecting the Humanitarian Health and Nutrition Response for Women and Children in Somalia Since 2000: A Case Study,” Conflict and Health 14 (2020): 35, 10.1186/s13031-019-0241-x. 32514300 PMC7254682

[17] A. A. Tahlil , O. M. Mohamud , S. M. Aden , et al., “Health‐Seeking Behavior in Conflict‐Affected Settings: A Cross‐Sectional Study of Internally Displaced Persons in Somalia,” Conflict and Health 19 (2025): 76, 10.1186/s13031-025-00718-5. 41084015 PMC12519811

[18] J. Morrison and S. Malik , “Health Equity in Somalia? An Evaluation of the Progress Made From 2006 to 2019 in Reducing Inequities in Maternal and Newborn Health,” International Journal for Equity in Health 23 (2024): 46, 10.1186/s12939-023-02092-1. 38443921 PMC10916226

[19] M. M. Hassan , A. N. Ali , I. Ali , et al., “Regulation of Health Professions Education and the Growth of Schools in Somalia,” BMC Medical Education 24 (2024): 1178, 10.1186/s12909-024-06179-3. 39427191 PMC11491018

[20] V. Percival , E. Dusabe‐Richards , H. Wurie , J. Namakula , S. Ssali , and S. Theobald , “Are Health Systems Interventions Gender Blind? Examining Health System Reconstruction in Conflict Affected States,” Global Health 14 (2018): 90, 10.1186/s12992-018-0401-6. 30157887 PMC6116483

[21] J. Morrison and S. Malik , “Population Health Trends and Disease Profile in Somalia 1990–2019, and Projection to 2030: Will the Country Achieve Sustainable Development Goals 2 and 3?,” BMC Public Health [Electronic Resource] 23 (2023): 66, 10.1186/S12889-022-14960-6. 36627611 PMC9832660

[22] M. G. Shah , T. Dey , and S. M. Kostelecky , “Guidance on Sexual, Reproductive, Maternal, Newborn, Child and Adolescent Health in Humanitarian and Fragile Settings: A Scoping Review,” BMJ Global Health 9 (2024): e013944, 10.1136/bmjgh-2023-013944. PMC10982774 38553049

[23] S. A. Hussein , M. M. Osman , M. M. Hassan , et al., “Combating Infectious Disease Outbreaks in Somalia's Fragile Health System: The Impact of Climate Change‐Narrative Review,” Tropical Medicine and Health 53 (2025): 142, 10.1186/S41182-025-00816-3. 41131623 PMC12548201

[24] A. J. Seal , M. Jelle , C. S. Grijalva‐Eternod , H. Mohamed , R. Ali , and E. Fottrell , “Use of Verbal Autopsy for Establishing Causes of Child Mortality in Camps for Internally Displaced People in Mogadishu, Somalia: A Population‐Based, Prospective, Cohort Study,” Lancet Global Health 9 (2021): E1286–E1295, 10.1016/S2214-109X(21)00254-0. 34416214

[25] S. C. Okoroafor , J. A. Asamani , L. Kabego , et al., “Preparing the Health Workforce for Future Public Health Emergencies in Africa,” BMJ Global Health 7 (2022): e008327, 10.1136/bmjgh-2021-008327. PMC9006823 35414522

[26] M. H. Adam , B. Garba , H. A. Dahie , et al., “Community‐Based Mortality Surveillance Among Internally Displaced Vulnerable Populations in Banadir Region, Somalia, 2022–2023,” Frontiers in Public Health 13 (2025): 1582558, 10.3389/FPUBH.2025.1582558. 40270735 PMC12014603

[27] V. Marou , C. I. Vardavas , K. Aslanoglou , et al., “The Impact of Conflict on Infectious Disease: A Systematic Literature Review,” Conflict and Health 18 (2024): 27, 10.1186/s13031-023-00568-z. 38584269 PMC11000310

[28] N. S. Singh , A. Ataullahjan , K. Ndiaye , et al., “Delivering Health Interventions to Women, Children, and Adolescents in Conflict Settings: What Have We Learned From Ten Country Case Studies?,” Lancet 397 (2021): 533–542, 10.1016/S0140-6736(21)00132-X. 33503459

[29] T. Dey , M. G. Shah , A. Baba , et al., “Reproductive, Maternal, Newborn, Child and Adolescent Health Services in Humanitarian and Fragile Settings: A Mixed Methods Study of Midwives' and Women's Experiences,” PLoS Global Public Health 4 (2024): e0003384, 10.1371/journal.pgph.0003384. 38959267 PMC11221643

[30] V. Onama and G. Babughirana , “Access and Utilization of Maternal Newborn and Child Health Services in the Fragile Context of Somalia,” Asploro Journal of Biomedical and Clinical Case Reports 6 (2023): 146–155, 10.36502/2023/ASJBCCR.6307.

[31] M. Mercogliano , G. Spatari , C. Noviello , et al., “Building Evidences in Public Health Emergency Preparedness (“BePHEP” Project)—A Systematic Review,” International Journal for Equity in Health 24 (2025): 41, 10.1186/s12939-025-02382-w. 39934889 PMC11817627

[32] Y. H. Abdi , M. S. Abdi , S. G. Bashir , N. I. Ahmed , and Y. B. Abdullahi , “The State of Public Health in Somalia: Top 5 Challenges and Strategies for Improvement,” Public Health Challenges 4 (2025): e70138, 10.1002/PUH2.70138. 41080794 PMC12509034

[33] A. J. Seal , H. A. Mohamed , and R. Stokes‐Walter , “Use of an Adapted Participatory Learning and Action Cycle to Increase Knowledge and Uptake of Child Vaccination in Internally Displaced Persons Camps (IVACS): A Cluster‐Randomised Controlled Trial,” Vaccine 41 (2023): 3038–3046, 10.1016/j.vaccine.2023.02.016. 36906409

[34] A. Gatome‐Munyua , S. Sparkes , G. Mtei , et al., “Reducing Fragmentation of Primary Healthcare Financing for More Equitable, People‐Centred Primary Healthcare,” BMJ Global Health 10 (2025): e015088, 10.1136/bmjgh-2024-015088. PMC11749059 39809525

[35] A. Y. Nuh and A. Y. Nuh , “Residence Inequality of Maternal Health Service Utilization Among Women in Somaliland: An Analysis From the Somaliland Demographic Health Survey in 2020,” Journal of Comprehensive Health 13 (2025): 89–95, 10.25259/jch_59_2024.

[36] K. Lindvall , J. Kinsman , A. Abraha , et al., “Health Status and Health Care Needs of Drought‐Related Migrants in the Horn of Africa—A Qualitative Investigation,” International Journal of Environmental Research and Public Health 17 (2020): 1–18, 10.3390/ijerph17165917. PMC7459765 32824046

[37] D. Cantor , J. Swartz , B. Roberts , et al., “Understanding the Health Needs of Internally Displaced Persons: A Scoping Review,” Journal of Migration and Health 4 (2021): 100071, 10.1016/j.jmh.2021.100071. 34820657 PMC8600058

[38] A. A. Mohamed , T. Bocher , and M. A. Magan , “Experiences From the Field: A Qualitative Study Exploring Barriers to Maternal and Child Health Service Utilization in IDP Settings Somalia,” International Journal of Women's Health 13 (2021): 1147–1160, 10.2147/IJWH.S330069. PMC8630365 34858064

[39] R. Walker , J. Vearey , A. S. Bile , and G. L. Lolimo , “Upholding the Right to Health in Contexts of Displacement: A Whole‐of‐Route Policy Analysis in South Africa, Kenya, Somalia, and the Democratic Republic of Congo,” International Journal of Environmental Research and Public Health 22 (2025): 1042, 10.3390/ijerph22071042. 40724109 PMC12294776

[40] S. Halane , A. M. Ahmed , M. M. Ahmed , et al., “Barriers and Disparities in Maternal and Child Vaccination Coverage in Galmudug State, Somalia: A Descriptive Study,” Sage Open Pediatrics 12 (2025): 30502225251388171, 10.1177/30502225251388171. 41181337 PMC12579158

[41] H. N. Njiru , P. Relan , S. Malik , et al., “Emergency and Critical Care Services in Somalia: A Cross‐Sectional Nationwide Hospital Assessment Using the WHO Hospital Emergency Unit Assessment Tool,” BMC Emergency Medicine 25 (2025): 89, 10.1186/S12873-025-01234-8. 40457224 PMC12131834

[42] M. M. Ahmed , “Revolutionizing Healthcare in Somalia: The Role of Digital Innovations in Enhancing Access and Quality,” Open Exploration 2 (2024): 360–368, 10.37349/edht.2024.00034.

[43] A. Endalamaw , R. B. Khatri , D. Erku , et al., “Barriers and Strategies for Primary Health Care Workforce Development: Synthesis of Evidence,” BMC Primary Care 25 (2024): 99, 10.1186/s12875-024-02336-1. 38539068 PMC10967164

[44] O. O. Ojo , O. O. Hersi , A. A. Falobi , N. Ali , L. Tan , and Y. A. Ali , “Assessment of the Capacity of Health Facilities in Preventing and Managing Non‐Communicable Diseases in Selected Regions of Somaliland,” BMC Health Services Research 25 (2025): 744, 10.1186/s12913-025-12913-4. 40410859 PMC12100831

[45] H. A. Abdi , M. O. Warsame , M. A. Adan , and M. A. Hassan , “Assessment of Patient Satisfaction Attaining Primary Health Care Services at Health Centers in Mogadishu, Somalia,” Patient Prefer Adherence 18 (2024): 2529–2543, 10.2147/PPA.S486919. 39687408 PMC11648542

[46] M. Ibrahim , H. Rizwan , M. Afzal , and M. R. Malik , “Mental Health Crisis in Somalia: A Review and a Way Forward,” International Journal of Mental Health Systems 16 (2022): 12, 10.1186/s13033-022-00525-y. 35139873 PMC8827242

[47] M. A. Jama , R. Majdzadeh , T. Reynolds , et al., “Revising the Essential Package of Health Services Through Stakeholder Alignment, Somalia,” Bulletin of the World Health Organization 101 (2023): 738–742, 10.2471/BLT.23.289733. 37961055 PMC10630727

[48] R. Baltussen , O. Mwalim , K. Blanchet , et al., “Decision‐Making Processes for Essential Packages of Health Services: Experience From Six Countries,” BMJ Global Health 8 (2023): e010704, 10.1136/bmjgh-2022-010704. PMC9853142 36657809

[49] A. Alwan , R. Majdzadeh , G. Yamey , et al., “Country Readiness and Prerequisites for Successful Design and Transition to Implementation of Essential Packages of Health Services: Experience From Six Countries,” BMJ Global Health 8 (2023): e010720, 10.1136/bmjgh-2022-010720. PMC9853149 36657808

[50] K. M. Mohamud , “Equity in Health Funding: An Analysis of the Essential Package of Health Services (EPHS) in Somalia (2021–2026),” Discover Health Systems 4 (2025): 132, 10.1007/S44250-025-00302-X.

[51] R. J. Montgomery , E. Scudder , C. Tulloch , M. Jama , N. Kozuki , and B. Ata , “Constrained Optimization: Evaluating Possible Packages of Community Health Interventions With Competing Resource Requirements in Galmudug, Somalia,” Health Policy and Planning 40 (2025): 566–577, 10.1093/heapol/czaf014. 40066997 PMC12063593

[52] D. A. Y. Ahmed , “Health Systems Governance in Somalia: An Examination of Validity, Digital Accountability, and Community Health Workforce Through Mixed Methods Research,” Advances in Social Sciences and Management 3 (2025): 138–167, 10.63002/assm.304.1056.

[53] A. T. Gebremeskel , O. Omonaiye , and S. Yaya , “Multilevel Determinants of Community Health Workers for an Effective Maternal and Child Health Programme in Sub‐Saharan Africa: A Systematic Review,” BMJ Global Health 7 (2022): e008162, 10.1136/bmjgh-2021-008162. PMC8991040 35393287

[54] T. A. Zerfu , M. Asressie , A. A. Tareke , et al., “Contributions of District Health Information Software 2 (DHIS2) to Maternal and Child Health Service Performance in Ethiopia: An Interrupted Time Series Mixed‐Methods Study,” Archives of Public Health 83 (2025): 173, 10.1186/s13690-025-01641-0. 40605114 PMC12220602

[55] T. A. Zerfu , M. Asressie , Z. Begna , et al., “Unveiling the Role of DHIS2 in Enhancing Data Quality and Accessibility in Primary Healthcare Facilities: Evidence From Ethiopia,” PLoS ONE 19 (2024): e0314505, 10.1371/journal.pone.0314505. 39693314 PMC11654967

[56] S. Siddiqi , W. Aftab , A. Venkat Raman , A. Soucat , and A. Alwan , “The Role of the Private Sector in Delivering Essential Packages of Health Services: Lessons From Country Experiences,” BMJ Global Health 8 (2023): e010742, 10.1136/bmjgh-2022-010742. PMC9853132 36657810

[57] K. Danforth , A. M. Ahmad , K. Blanchet , et al., “Monitoring and Evaluating the Implementation of Essential Packages of Health Services,” BMJ Global Health 8 (2023): e010726, 10.1136/bmjgh-2022-010726. PMC10069525 36977532

[58] D. Scharff , K. R. Enard , D. Tao , G. Strand , R. Yakubu , and V. Cope , “Community Health Worker Impact on Knowledge, Antenatal Care, and Birth Outcomes: A Systematic Review,” Maternal and Child Health Journal 26 (2022): 79–101, 10.1007/s10995-021-03299-w. 34981332

[59] T. Simbini , E. Adimado , S. Adjorlolo , et al., “Digital Health Interventions (DHIs) for Health Systems Strengthening in Sub‐Saharan Africa: Insights From Ethiopia, Ghana, and Zimbabwe,” preprint, MedRxiv, April 25, 2025, 10.1101/2025.04.22.25326213. PMC12758792 41481760

[60] K. Hanson , “Introducing The Lancet Global Health Commission on Financing Primary Health Care: Putting People at the Centre,” Lancet Global Health 10 (2022): E20–E21, 10.1016/S2214-109X(21)00510-6. 34919847

[61] A. Edelman , R. Marten , H. Montenegro , et al., “Modified Scoping Review of the Enablers and Barriers to Implementing Primary Health Care in the COVID‐19 Context,” Health Policy and Planning 36 (2021): 1163–1186, 10.1093/heapol/czab075. 34185844 PMC8344743

[62] P. B. Kodali , “Achieving Universal Health Coverage in Low‐ and Middle‐Income Countries: Challenges for Policy Post‐Pandemic and Beyond,” Risk Management and Healthcare Policy 16 (2023): 607–621, 10.2147/RMHP.S366759. 37050920 PMC10084872

[63] A. Debie , A. Nigusie , D. Gedle , R. B. Khatri , and Y. Assefa , “Building a Resilient Health System for Universal Health Coverage and Health Security: A Systematic Review,” Global Health Research and Policy 9 (2024): 2, 10.1186/s41256-023-00340-z. 38173020 PMC10765832

[64] R. B. Khatri , A. Endalamaw , D. Erku , et al., “Contribution of Health System Governance in Delivering Primary Health Care Services for Universal Health Coverage: A Scoping Review,” PLoS ONE 20 (2025): e0318244, 10.1371/journal.pone.0318244. 40019911 PMC11870385

[65] R. R. Basapathy and M. R. Mathur , “Strengthening Governance for Universalising Primary Oral Health Care: Perspectives From Karnataka, India,” Wellcome Open Research 10 (2025): 305, 10.12688/wellcomeopenres.24152.1. 41208825 PMC12595295

[66] S. M. Tarekegn , D. Tadesse , M. D. Argaw , et al., “Capacity and Performance of Primary Health Care in Ethiopia: A Novel Mixed Methods Measurement in Low‐Income Country,” BMC Primary Care 26 (2025): 299, 10.1186/s12875-025-02988-7. 41023838 PMC12481810

[67] N. A. Pradhan , A. Samnani , K. Abbas , and N. Rizvi , “Resilience of Primary Healthcare System Across Low‐ and Middle‐Income Countries During COVID‐19 Pandemic: A Scoping Review,” Health Research Policy and Systems 21 (2023): 98, 10.1186/s12961-023-01031-4. 37723533 PMC10507852

[68] S. A. Hussein , M. M. Osman , Y. S. A. Hassan , et al., “Strengthening Somalia's Health System: Pathways to Achieving International Health Regulations Core Capacities at Points of Entry by 2025,” Tropical Medicine and Health 53 (2025): 159, 10.1186/S41182-025-00836-Z. 41214756 PMC12604186

[69] D. S. Saminarsih , C. C. Magdalena , N. Febianisari , et al., “Synthesising Key Findings From Indonesia's First Civil Society‐Led Forum on Primary Health Care: PHC Forum, Jakarta, 13–14 November 2023,” BMC Proceedings 19 (2025): 21, 10.1186/s12919-025-00338-0. 40830870 PMC12366000

[70] A. Massuda , M. Fernandez , M. A. C. Paschoalotto , and E. S. Kemper , “Primary Health Care Policy Investments in the Latin America Context: Health Systems Experiences From Brazil, Chile, and Colombia,” Health Policy Open 9 (2025): 100147, 10.1016/j.hpopen.2025.100147. 40917759 PMC12409964

[71] G. S. Adithyan , A. Ranjan , V. R. Muraleedharan , and T. Sundararaman , “Kerala's Progress Towards Universal Health Coverage: The Road Travelled and Beyond,” International Journal for Equity in Health 23 (2024): 152, 10.1186/s12939-024-02231-2. 39103907 PMC11302021

[72] E. C. Langat , P. Ward , H. Gesesew , and L. Mwanri , “Challenges and Opportunities of Universal Health Coverage in Africa: A Scoping Review,” International Journal of Environmental Research and Public Health 22 (2025): 86, 10.3390/ijerph22010086. 39857539 PMC11764768

[73] A. Edelman , L. Vinyals Torres , A. Kazi , K. Rasanathan , and R. Marten , “An Unfinished Agenda: Insights From Seven Country Case Studies on Strengthening Primary Health Care in the Western Pacific Region,” BMJ Global Health 10 (2025): 1–8, 10.1136/bmjgh-2024-017442. PMC11969583 40174966

[74] S. B. Rifkin , “Alma Ata After 40 Years: Primary Health Care and Health for All‐From Consensus to Complexity,” BMJ Global Health 3 (2018): e001188, 10.1136/bmjgh-2018-001188. PMC6307566 30622747

[75] E. V. Langlois , A. McKenzie , H. Schneider , and J. W. Mecaskey , “Measures to Strengthen Primary Health‐Care Systems in Low‐ and Middle‐Income Countries,” Bulletin of the World Health Organization 98 (2020): 781–791, 10.2471/BLT.20.252742. 33177775 PMC7607465

[76] N. Joudyian , L. Doshmangir , M. Mahdavi , J. S. Tabrizi , and V. S. Gordeev , “Public‐Private Partnerships in Primary Health Care: A Scoping Review,” BMC Health Services Research 21 (2021): 4, 10.1186/s12913-020-05979-9. 33397388 PMC7780612

[77] L. A. Omam , E. Jarman , K. N. O'Laughlin , and R. Parkes‐Ratanshi , “Primary Healthcare Delivery Models in African Conflict‐Affected Settings: A Systematic Review,” Conflict and Health 17 (2023): 34, 10.1186/s13031-023-00533-w. 37454133 PMC10349495

[78] L. A. Omam , K. O'Laughlin , and N. Tendongfor , “Exploring Factors Influencing the Selection of Primary Health Care Delivery Models in Conflict‐Affected Settings of North West and South West Regions of Cameroon and North‐East Nigeria: A Study Protocol,” PLoS ONE 18 (2023): e0284957, 10.1371/journal.pone.0284957. 37134075 PMC10155952

[79] B. Bogale , S. Scambler , A. N. Mohd Khairuddin , and J. E. Gallagher , “Health System Strengthening in Fragile and Conflict‐Affected States: A Review of Systematic Reviews,” PLoS ONE 19 (2024): e0305234, 10.1371/journal.pone.0305234. 38875266 PMC11178226

